# Comprehensive Overview of Quality of Life Instruments Used in Studies of Children with Diabetes: A Systematic Mapping Review

**DOI:** 10.1155/2023/8885973

**Published:** 2023-09-25

**Authors:** Louise Norman Jespersen, Tue Helms Andersen, Dan Grabowski

**Affiliations:** ^1^Department of Health Promotion Research, Diabetes Management, Copenhagen University Hospital – Steno Diabetes Center Copenhagen, Borgmester Ib Juuls vej 83, Herlev 2730, Denmark; ^2^Department of Education, Danish Diabetes Knowledge Center, Copenhagen University Hospital – Steno Diabetes Center Copenhagen, Borgmester Ib Juuls vej 83, Herlev 2730, Denmark

## Abstract

**Background:**

Quality of life (QoL) is extensively used as an outcome in the studies of children with diabetes. The interest in measuring QoL in relation to clinical treatment and interventions has led to an increase in the development and use of QoL measures. The vast number of available instruments can be a barrier for establishing evidence and can be overwhelming for clinicians and researchers who are interested in measuring QoL of children with diabetes.

**Aim:**

As a first step for reaching consensus, we aimed to provide a comprehensive overview of the application of QoL instruments used in children (2–18 years old) with diabetes.

**Method:**

A literature search for studies published from inception to January 2022 was conducted in the databases MEDLINE (Ovid), Embase (Ovid), PsycInfo (EBSCO), CINAHL (EBSCO), and ERIC (EBSCO). The search strategy combined the key concepts of “quality of life”, “diabetes”, and “children or adolescents”. Studies were found eligible if (1) the population was below 19 years of age; (2) had diabetes mellitus; and (3) a quantitative measure of QoL was used.

**Results:**

3,775 unique articles were retrieved in the literature search and, across 503 articles included for synthesis, 67 QoL instruments were identified. The instruments were classified by i.a. population age, continent, use of pre–post measure, self-report or proxy, and type of diabetes.

**Conclusion:**

The extensive number of QoL instruments that are used for children with diabetes constitutes a substantial barrier for establishing evidence in relation to QoL in this research area.

## 1. Introduction

Quality of life (QoL) is widely recognized as an important health outcome in the treatment of pediatric diabetes [1, 2]. The complex and constant illness management required during childhood and adolescence places a significant burden on children with diabetes and their families. This heavy burden can lead to serious psychosocial problems, which may affect diabetes management, and ultimately lead to dysregulated blood glucose in the children [3, 4]. Systematic monitoring of QoL has been shown to improve adolescents' well-being and satisfaction with care [5] and, if used appropriately, regular measures of QoL can identify children with reduced QoL, and hence guide timely interventions to help the children enhance their QoL [6]. It has been suggested that regular assessment of the family's psychosocial needs, and subsequent interventions addressing potential challenges, may be as important to diabetes management as insulin, diet, and physical activity [7]. Furthermore, The International Society for Pediatric and Adolescent Diabetes (ISPAD) guidelines state that pediatric diabetes teams should routinely assess the QoL of their patients [8] and that the shared goal of care should be: “*to optimize health outcomes and health related quality of life*” [8]. Regular measures of the QoL in children with diabetes can be critical for decision-making in the pediatric diabetes clinic as a valuable supplement to clinical and biological measures, for example, when deciding which treatment option is best for the child and his/her family [9, 10].

In studies of children and adolescents with diabetes, QoL is extensively used as a primary and secondary outcome [11]. More than 20 years ago, Polonsky [12] pointed out that interest in measuring QoL grew at a phenomenal rate during the 80s and 90s. He further stressed that poor definitions of QoL and the number of instruments purporting to measure the concept were overwhelming [12]. Since then, continued use of QoL as a primary or secondary outcome has led to a further increase in the development and use of QoL measures [10]. For those who do not have a good understanding of how to select an appropriate QoL measure, the vast number of available questionnaires claiming to measure QoL in children with diabetes may be overwhelming [2] and become a barrier for clinicians and researchers interested in acquiring knowledge about QoL in children with diabetes [13]. For example, in a recent review on health-related QoL in children with diabetes using insulin infusion systems, Rosner and Roman-Urrestarazu [14] identified eight different instruments across the 15 included studies. The authors claimed that the large heterogeneity in assessment of QoL and differences in how it was reported limited their meta-analysis [14].

Because the concept of QoL is complex, subjective, and thus not directly observable, it is undisputedly difficult to quantify. Unfortunately application of different terms to describe one concept, or of one term to describe different concepts, is a contributing factor to the continued development of questionnaires claiming to measure QoL in children [6]. Furthermore, many questionnaires have been developed without the use of appropriate methods or best practices [15]. There is still no consensus in the definition or measurement of QoL among children with diabetes, and few research papers have stated their definition of QoL or their rationale for choosing a specific questionnaire [12]. This lack of attention regarding the choice of QoL instrument may have huge implications for the research area because: “*You can't fix by analysis what you've spoiled by design*” [15], meaning that the instrument defines the data on which the analyses will be based. This may imply that the data collected through inappropriate instruments are not reproducible or, in the worst case, fail to measure the essence of the concept the researchers expect the instrument to capture [15]. Ultimately, these issues can impact the reliability and validity of survey data, making it difficult to trust any conclusions drawn.

As a necessary first step for establishing evidence on QoL for children with diabetes, this systematic mapping review will serve as a comprehensive starting point for future consensus on the QoL instruments. By conducting a thorough literature search, screening, and classification of journal articles reporting on QoL in children with diabetes, the present systematic mapping review aims to provide a comprehensive overview of the instruments used for measuring QoL in children (2–18 years old) with diabetes.

## 2. Methods

Because the aim of this present paper was to create an overview of scientific literature measuring QoL among children and adolescents with diabetes, a systematic mapping approach was used. Booth [16] explains that mapping reviews are especially useful for areas in which a large volume of literature exists. Furthermore, that systematic mapping reviews are useful to conduct prior to more narrow systematic reviews since they can be used to identify knowledge gaps. Mapping reviews can thus be used to code and categorize an extensive body of literature, at a more general level, and subsequently be used for guidance when selecting more narrow themes for detailed systematic reviews [17].

The reporting in the present systematic mapping review follows the Preferred Reporting Items for Systematic Reviews and Meta-Analyses (PRISMA) extension for scoping reviews (PRISMA-ScR) adapted to a mapping review [16, 18]. The reporting of the literature search follows the PRISMA Statement for Reporting Literature Searches in Systematic Reviews (PRISMA-S) [19].

### 2.1. Eligibility Criteria

Studies were found eligible if (1) the population (or a major part of the population) was below 19 years of age; (2) had diabetes mellitus; and (3) a quantitative measure of QoL was used.

There is substantial debate around the construct of QoL and the conceptual approach across the included studies and instruments is most likely to vary. However, we wanted to include all studies adding to the evidence on QoL for children with diabetes and thus did not limit our search to a specific conceptual approach. Because of the indiscriminate use of QoL and health-related quality of life (HRQoL), we included both terms to avoid missing important studies.

Studies were excluded if they were not journal articles including primary research data, for example, reviews, editorials, discussion points, conference abstracts, and book chapters.

### 2.2. Search Strategy

An electronic literature search for studies published from inception to 13 February 2020 was conducted in the databases MEDLINE (Ovid), Embase (Ovid), PsycInfo (EBSCO), CINAHL (EBSCO), and ERIC (EBSCO) (Supplementary 1). The search was updated in all five databases on January 18, 2022. The search strategy combined the key concepts of “quality of life,” “diabetes,” and “children or adolescents.” We searched all concepts using both relevant medical subject headings, multiple free text words, and keywords where possible.

We applied a validated filter removing animal studies in MEDLINE and Embase. Because no validated filter was available for PsycInfo, CINAHL, and ERIC, we decided not to take the risk of applying filters in those databases. An “exclude MEDLINE journals” limit was applied in Embase and CINAHL to remove references already captured in the MEDLINE search.

The search strategy was developed and tested in MEDLINE to evaluate if the search string retrieved known key articles of interest. Subsequently, we translated the search strategy to the other databases.

All identified records were uploaded to and organized in EPPI Reviewer Web, a web application developed to conduct systematic reviews [20]. Two information specialists conducted the literature search, and the entire search strategy is documented in Supplementary 1.

### 2.3. Study Selection

Deduplication was performed in EPPI Reviewer Web in two steps. First, an automatic function identified possible duplicates, and one author verified and accepted all true duplicates (*n* = 616). Second, all remaining records were manually examined by one author to detect duplicates not already identified by the automatic duplicate function (*n* = 54).

All records were screened independently by title and abstract by two researchers according to the eligibility criteria. Disagreements were solved by consensus and, if not possible, subsequently by a third researcher. Full-text reports of all included records were first sought for retrieval electronically. Second, we attempted to retrieve the records physically from the Danish Royal Library. Third, an e-mail was sent to the first author of the record. If no answer was received after 1 month, then the record was excluded.

Records in other languages than English, Danish, Swedish, and Norwegian (languages spoken in the review team) were not included in the synthesis, but are listed in supplementary materials for others to analyze (see Supplementary 2).

### 2.4. Quality Assessment

In accordance with guidelines on systematic mapping reviews [16], no quality assessment of the 503 included studies was conducted.

### 2.5. Data Extraction and Synthesis

Data extraction was conducted according to a mapping strategy aiming to classify included studies under the following themes: “instrument(s) used”, “use of pre–post measure,” “continent,” “population age,” “population size,” “telephone, internet or off-line,” “interview or self-complete,” “self-report or proxy,” and “type of diabetes.” Furthermore, we extracted information on the title, author, and year of publication. All data were extracted by one researcher and subsequently verified by another. Disagreements were discussed, if needed with a third researcher, and consensus was reached. The synthesis was conducted with a visualization and mapping function in EPPI Reviewer Web to create an evidence gap map [21].

## 3. Results

Interrater reliability (IRR) for the title and abstract screening was 92.776% and for full-text screening IRR was 92.780%.

**Figure 1:**
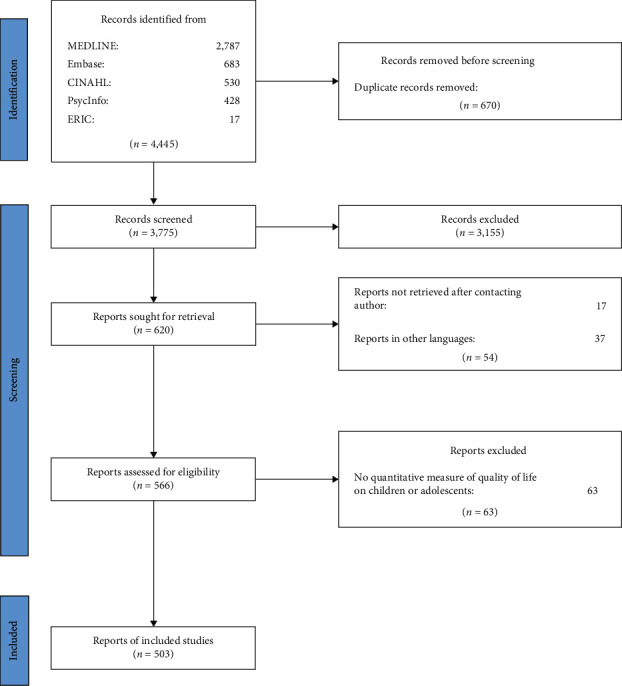
Flow diagram of the selection of articles on quality of life in children with diabetes.

The literature search returned a total of 4,445 papers, which were reduced to 3,775 after checking for duplicates. After screening the papers' titles and abstracts, 620 papers were included in the review. Additional screening of the full-text papers excluded 117 papers from the synthesis (Supplementary 2). Thirty-seven were written in a language other than English, Danish, Swedish, or Norwegian, 63 did not include a quantitative measure of QoL in children or adolescents with diabetes and 17 were excluded because we could not access the full-text article after electronic search, library search, and direct contact to the first author. The flow diagram in Figure 1 illustrates the selection of articles.

**Figure 2:**
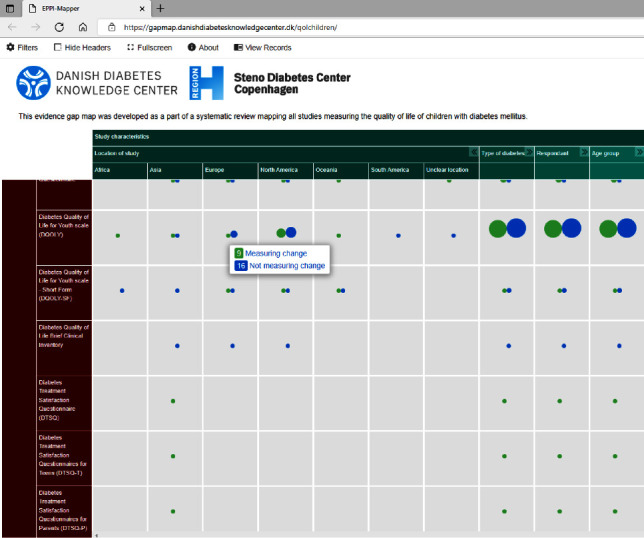
Screenshot of a selected area of the interactive evidence gap map available online.

To create transparency, visualize research available, illustrate gaps in the literature, and allow readers to further explore the data extracted in the present mapping review, an interactive evidence gap map with all included studies was created and made accessible at: https://gapmap.danishdiabetesknowledgecenter.dk/qolchildren. A screenshot of a selected area of the evidence gap map is shown in Figure 2.

**Figure 3:**
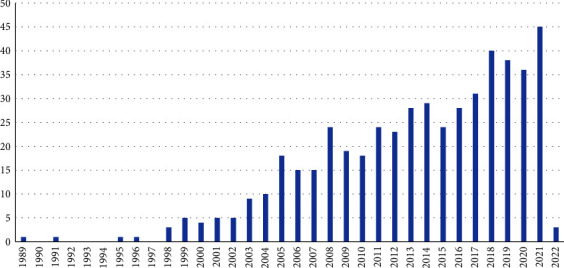
Distribution of included articles by publication year.

As illustrated in Figure 3, the articles were published between 1989 and 2022, with a steady rise in frequency from 1996 to 2021. Because the search was conducted in January 2022, only 3 articles from 2022 were included.

A total of 445 (88.5%) of the included articles targeted children with type-1 diabetes exclusively. Only 10 articles (2.0%) targeted children with type-2 diabetes exclusively, whereas 18 (3.6%) articles included children with type-1 or type-2 diabetes. Studies including other types of diabetes were coded as type-1 or type-2 according to which of those two types was the dominant.

Across the 503 included articles, a total of 67 different QoL instruments were identified. The 67 instruments include different versions of the same core questionnaire (e.g., versions for children, adolescents, or parent proxy).

A list of all included instruments is presented in Table 1. Where slightly different names were used to apparently describe the same instrument, the paper and references were scrutinized to confirm which instrument was used, to ensure that the correct instruments were registered in the review. An important finding was that, in 30 examples (6.0%), reports on QoL in children with diabetes were based on a questionnaire that we were not able to identify with certainty. In these instances, the instrument was coded as “self-constructed/unclear.” To highlight the instruments currently in use, instruments in Table 1 are written in italics if they have not been referred to in publications since 2016.

**Figure 4:**
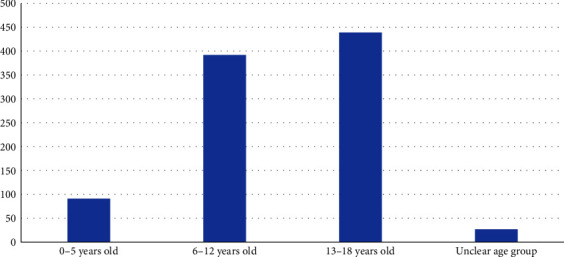
Distribution of articles by age of the target group.

To illustrate the use of instruments in different age groups, the age of the target population was categorized into three groups: <6 years, 6–12 years, and 13–18 years. If the target population included, for example, 8- to 15-year-olds, both groups (6–12 years, and 13–18 years) were selected. In some articles, age of the target group was indicated by mean and standard deviation (SD), but without indication of the age range. In other studies, age range was mentioned among the inclusion criteria, but not reported for the actual study population. In these instances, and where no age was reported, age was coded as “unclear.” The distribution of age groups is illustrated in Figure 4, showing that most research on QoL in children with diabetes is conducted within the age range of 13- to 18-year-olds, whereas studies on QoL in the youngest children are sparse, with fewer than 100 studies in total. In 25 articles, we were not able to identify the precise age range of the target population.

**Figure 5:**
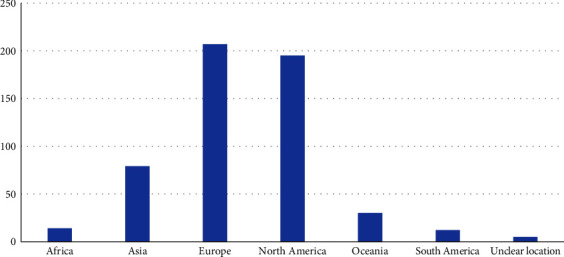
Distribution of articles by continent.

As shown in Figure 5, the vast majority of studies on QoL in children with diabetes were conducted in Europe (207) and North America (195), whereas only 30, 14, and 12 studies were conducted in Oceania, Africa, and South America, respectively. Studies conducted in Turkey or Russia were coded as both “Asia” and “Europe.”

Furthermore, we found that some instruments tended to be used exclusively on the continent in which they were originally developed (data can be retrieved from the Evidence Gap Map). For example, the DISABKIDS, which was developed based on a sample from seven European countries [40], was reported a total of 50 times, but only three of the cases were from studies conducted outside Europe. The only instruments that have been used across all continents were DQOLY [32] and PedsQL [72].

Of the 67 identified instruments, 43 (64.2%) have been used, across 206 articles, to measure change in QoL by comparing measures before and after a treatment procedure or intervention. Especially DQOLY [32] and PedsQL [72] were popular choices for measuring change in children's QoL.

Although most of the articles used instruments to measure QoL as experienced by the children themselves (self-report), 35.4% included both a self-report and a proxy measure, whereas 6.4% used only a proxy measure to quantify the children's QoL.

The ten instruments most frequently used to measure QoL among children with diabetes are: PedsQL (nonspecific), PedsQL Generic Core Scale, PedsQL Diabetes Module, DQOL, DQOLY, DQOLY-SF, DISABKIDS, DISABKIDS Generic Module Short Form, DISABKIDS Disease-Specific Module for Diabetes, and KINDL. A table with key characteristics of the instruments most frequently used was made to illustrate differences and similarities (Table 2). Since the nonspecific PEDSQL must be either the Generic Core Scale or the Diabetes Module, only nine measures were included in the table.

## 4. Discussion

Across the 503 papers included in the present systematic mapping review, we identified 67 different instruments that have been used to measure QoL in children with diabetes. For an additional 30 instruments we were not able to identify the instrument with certainty, and in 25 of the included articles, we could not identify the precise age range of the target group. These findings emphasize the importance of precise reporting of the target population and the instruments used. In accordance with previous research [12], it seemed as though rationales for selecting a particular questionnaire were often missing and that the most frequently used rationales were that it was: (1) widely used and (2) validated (data not shown).

Because different research questions call for different characteristics in a QoL instrument, selection of such an instrument should always be guided by the research question being investigated [12]. However, the large number of available instruments identified in this review may add to the challenge faced by researchers and clinicians, in that limited time and resources may cause them to choose a measure that is less than optimal [73]. While recognizing that “the single best” QoL instrument for children with diabetes does not exist in an absolute sense, there are instruments that are better choices for specific purposes [74], and there are several things to consider when searching for the most suitable instrument for measuring QoL in children with diabetes.

Based on the screening of articles for the present systematic mapping review, we recognized a need for emphasizing the importance of including a section on the applied instruments in all research articles. To select the most appropriate instrument, awareness of the reason for measuring QoL is crucial. Therefore, a deliberate rationale for wanting to measure QoL should be considered before deciding on an instrument; and the rationale for using the selected instrument should always be provided in research articles to help readers evaluate the validity of the results [75]. The fact that the instrument is widely used and/or validated is not a sufficient argument. Furthermore, the section should include a thorough description of the instruments on which the results are based. Sometimes the instrument is mentioned using a slightly modified name or simply just described as “a quality of life questionnaire,” which makes it impossible for the readers to identify the instrument used [75]. It is crucial to provide the precise name and details and to include a correct reference, so that the original instrument can be identified.

In the following, we will expand on the common “widely used” and “validated” arguments by accompanying our results with some general considerations about QoL instrument selection for children with diabetes.

### 4.1. The Developing Concept of QoL

Because the time since development of the instrument may partially explain its frequency of use, appropriateness for the target population should always be considered—even if the instrument is widely used and validated. Obviously, the dissemination and validation of more recently developed instruments do not compare with instruments developed decades ago. Nevertheless, newer instruments may be based on a more updated approach to QoL and thus provide a more contemporary reflection of the concept. The most frequently used instruments in the present review were originally developed 17–30 years ago [32, 72, 76], and although our results illustrate how the original instruments have been adapted and adjusted to suit different needs, for example, a version specifically suitable for children and adolescents with diabetes [59], the rationale behind some of the most frequently used QoL assessments may not be in accordance with the most recent conceptual understanding. For example, a review of QoL measures for adults with diabetes (2020) underlined the importance of considering the surrounding environment and society as part of a QoL assessment, and the authors described how failing to do so may entail missing information on perceptions of QoL related to, for example, a supportive health care system and societal attitude toward illness [10]. This more recent approach to QoL measurement suggests that QoL should not only be reduced to a measure of the absence of negative aspects related to the illness, but also include aspects of psychological coping and acceptance [10]. Whereas many illness-specific instruments address potential negative aspects of life, few address aspects such as acceptance, hope, and having a positive attitude toward the illness [10].

### 4.2. Applicability to Other Target Groups

Another aspect to keep in mind, before deciding on an instrument, is the applicability of an existing instrument to a different target group. Results from the present mapping review showed that some instruments were mainly used on the continent where they were developed [76], whereas others have been used across all continents [32]. Some instruments were developed across a number of countries [40], whereas others were developed in a specific cultural setting [32]. When instruments are applied in a different cultural setting than they were originally developed for, investigation of their cultural applicability and adaptation to the new context is necessary. Adaptation to a different context is often reduced to a translation from the source language to a target language [77]. The quality of such an instrument translation varies, but even when a careful forward–backward translation procedure is conducted, a linguistic translation does not guarantee that the instrument is culturally relevant [77]. Therefore, the original target group, for example, age and type of illness, cultural context, and what purpose the instrument was originally developed for should always be investigated and considered before deciding on an instrument. If using the instrument in a different target group or for a different purpose, feasibility tests should be conducted to ensure that the instrument is relevant to the new context [77].

### 4.3. Awareness of the Properties of Instruments

The present mapping review identified 206 articles using 43 different instruments used to measure change in QoL. When aiming to measure the impact of an intervention or treatment procedure on QoL in children with diabetes, an impact is more likely to be captured by a diabetes-specific measure rather than by a generic measure [12], because some of the items in a generic measure may be perceived as insignificant to children with diabetes. Insignificant items are known to be less sensitive to change because responses tend to cluster at the ends of the response scale [74]. Instruments are more likely to detect change in QoL if the responses of the target population are distributed around the middle of the response options. Thus, an instrument's ability to detect changes depends on the target population [74]. Unfortunately, the ability of instruments to detect chance over time (responsiveness to change) is often not properly investigated [78]. This means that changes in QoL can be difficult to detect, leading to erroneous conclusions.

Two overarching approaches, “generic” and “specific,” can be employed when assessing QoL among children with diabetes. For comparison of children with other illnesses, a chronic generic approach (i.e., including different chronic conditions) can be considered [41]. If a study aims to compare the QoL of children with diabetes to that of children without any chronic condition, a generic instrument should be used. Diabetes-specific instruments are not appropriate for comparison across target groups without diabetes because they address dimensions that are specifically relevant to the lives of children with diabetes, but may be irrelevant to children without diabetes (e.g., issues related to pain and stigma). Because of their different assessment of QoL, it is often suggested that both generic and diabetes-specific aspects of QoL should be included in studies of children with diabetes.

### 4.4. Implications for Research

Considering the challenges described in the present systematic mapping review, we should use due caution when (1) selecting an instrument; (2) citing an instrument; as well as (3) reporting and reading conclusions based on an instrument. Which concept is actually being measured? Is the applied instrument appropriate for the target group? Which properties do the applied instrument have? Despite this complexity, we should continue to strive to select sound and informative measures of QoL in children with diabetes.

Based on the overview created in this present study, narrow themes for detailed systematic reviews can be identified. Such reviews could for example focus on studies used within a specific age range, studies including a pre–post measure or studies using a specific QoL-instrument (data can be retrieved from the evidence gap map).

### 4.5. Implications for Practice

The present mapping review provides an overview of instruments used to measure QoL in children with diabetes. The overview and descriptions may increase awareness of the importance of selecting an appropriate QoL instrument and facilitate the process of selecting appropriate measures for use in pediatric diabetes clinics.

Due to the limited time resources, on the part of both professionals and children with diabetes, instruments with few items are often favored. Although this is understandable, our review underlines the importance of selecting an instrument suitable for addressing the question of interest. For example, applying an instrument that is not able to measure change over time can lead to the conclusion that an intervention had no effect on the children's QoL—disregarding the actual impact.

## 5. Conclusion

In conclusion, the present mapping review identifies and describes the extensive number of QoL instruments used for children with diabetes. The vast number of available instruments is a challenge when searching for the most appropriate instrument to include in a study, which further complicates comparison across studies. This disparity constitutes a substantial barrier for establishing evidence in relation to QoL in this research area.

## Figures and Tables

**Table 1:** Instruments identified in the systematic mapping review (instruments in italics have not been referred to in publications since 2016).

Instrument	Code ID	Count
*Audit of diabetes-dependent QoL (ADDQoL) [22]*	10243539	1
*Audit of diabetes-dependent QoL for teenagers (ADDQoL-Teen) [23]*	10243541	1
*Berner subjective well-being inventory (BFW)^NA^*	10243542	1
Check your health [24]	10243543	5
*Child behavior checklist (CBCL) [25]*	10243544	1
*Child health questionnaire (CHQ) [26]*	10243545	4
*Child health questionnaire - parent form 50 (CHQ-PF50) [27]*	10243546	4
*Child health questionnaire child form 80 (CHQ-CF80) [28]*	10243547	2
Child health questionnaire child form 87 (CHQ-CF87) *[26]*	10243548	5
*Child health & illness profile - child edition (CHIP-CE) [29]*	10243550	1
*Childhood Illness Scale (ICI) [30]*	10243552	1
Diabetes quality of life (DQOL) questionnaire [31]	10243555	22
Diabetes quality of life for youth scale (DQOLY) [32]	10243558	86
Diabetes quality of life for youth scale - short form (DQOLY-SF) [33]	10243559	15
Diabetes quality of life brief clinical inventory [34]	10243560	3
*Diabetes treatment satisfaction questionnaire (DTSQ) [35]*	10243561	1
Diabetes treatment satisfaction questionnaires for parents (DTSQ-parent) [36]	10243568	2
Diabetes treatment satisfaction questionnaires for teens (DTSQ-teen) [36]	10243569	2
*Diabetes-39 (D-39) [37]*	10974543	1
DISABKIDS [38]	10243570	10
DISABKIDS, chronic generic module, short version (DCGM-12) [39]	10243571	19
DISABKIDS, disease-specific module for diabetes (DM) [40]	10243572	15
DISABKIDS chronic generic module (DCGM-37) [41]	10243573	1
DISABKIDS, disease-specific module for diabetes, proxy version (DM - proxy) [41, 42]	10974544	1
DISABKIDS chronic generic module - long version - proxy version (DCGM-37 - proxy) [41]	10243574	4
EuroQol 5-dimension - youth version (EQ-5D-Y) [43]	10243579	11
EUROHIS-QOL 8-item index [44]	10982634	1
Ferrans and powers quality of life index^NA^	11012388	1
*Guidelines for adolescent preventive services (GAPS) questionnaire [45]*	10243580	1
Health-related quality of life questionnaire for diabetics^NA^	11012389	1
*Health utilities index (HUI®) [46]*	10243581	1
Smiley faces questionnaire [47]	10974545	2
Instrumento de qualidade de vida para jovens com diabetes (IQVJD) (Spanish)^NA^	11012645	1
*Insulin delivery system rating questionnaire (IDSRQ) [48]*	10243585	1
*Insulin treatment satisfaction questionnaire (ITSQ)*	10243586	1
*Iranian diabetes quality of life (IRDQOL) [49]*	10974541	1
KINDL questionnaire [50]	10243587	17
Kid-KINDL (8–12 years) [50]	10243589	3
Kiddo-KINDL (13–16 years) [50]	10243590	5
KINDL, parents version [50]	10243591	1
KINDL, diabetes module [50]	10243593	3
Kids-CAT [51]	10243594	5
KIDSCREEN [52]	10243596	1
KIDSREEN-10 (index) [53]	10243598	6
KIDSCREEN-27 (short version) [54]	10243599	8
KIDSCREEN-27 (proxy) [54]	10243600	6
*KIDSCREEN-52 (long version) [52]*	10243601	2
*Manchester-Minneapolis quality of life instrument (MMQL) child form [55]*	10243602	2
MIND youth questionnaire (MY-Q) [56]	10243603	10
*Oral health impact profile-14 (OHIP-14) [57]*	10243606	2
*Pediatric diabetes quality of life (PDQ)^NA^*	10243609	1
Pediatric quality of life inventory (PedsQL) [58]	10243613	58
Pediatric quality of life inventory (PedsQL) generic core scale [58]	10243614	97
Pediatric quality of life inventory (PedsQL) diabetes module [59]	10243615	126
Pediatric quality of life inventory (PedsQL) parent-proxy report [60]	10243616	3
*Pediatric quality of life inventory (PedsQL) adolescents [58]*	10243619	2
*Pediatric quality of life inventory (PedsQL) generic core scale short form 15 [61]*	10974542	1
General health survey, short form - SF-8 [62]	10982633	1
*General health survey, short form - SF-12 [63]*	10243621	1
*General health survey, short form - SF-36 [64]*	10243622	2
*Schedule for the evaluation of individual quality of life: a direct weighting procedure for quality of life domains (SEIQoL-DW) [65]*	10243625	2
*Self-perception profile for adolescents [66]*	11012148	1
*TACQOL - TNO-AZL questionnaire for children's health-related quality of Life [67]*	10974547	1
*TAPQOL - TNO-AZL questionnaire for preschool children's health-related quality of life [68]*	10243626	2
The self-report type-1 diabetes and life (T1DAL) measures for children and adolescents [69]	10243628	1
Vecu et sante percue de l'Adolescent (VSP-A) [70]	10243629	3
The 5-item world health organization well-being index (WHO-5) [71]	10243631	5
Self-constructed/unclear	10974549	30

**Table 2:** Key characteristics of the instruments most frequently used to measure quality of life among children with diabetes.

Instrument	Developed to measure	Year of development	Items	Domains	Response	Generic or specific	Available versions for children
Pediatric quality of life inventory (PedsQL) generic core scale [67]	Health-related quality of life	1998	23	Physical functioning Emotional functioning Social functioning School functioning	5-point Likert scale	Generic	*Self-report* 5–7 years 8–12 years 13–18 years *Proxy* 2–4 years 5–7 years 8–12 years 13–18 years

Pediatric quality of life inventory (PedsQL) diabetes module [68]	Diabetes-specific quality of life	2003	28 (3.0) 33 (3.2)	Diabetes Treatment Worry Communication	5-point Likert scale	Diabetes specific	*Self-report* 5–7 years 8–12 years 13–18 years *Proxy* 2–4 years 5–7 years 8–12 years 13–18 years

Diabetes quality of life (DQOL) questionnaire [43]	Patient-perceived personal burden of the DCCT trial	1988	46	Satisfaction Impact Diabetes worry Social/vocational worry	5-point Likert scale	Diabetes specific	*Self-report* 13–40 years

Diabetes quality of life for youth scale (DQOLY) [23]	Self-perceived quality of life	1991	52	Diabetes life satisfaction Disease impact Disease-related worries	5-point Likert scale	Diabetes specific	*Self-report* 11–22 years

Diabetes quality of life for youth scale—short form (DQOLY-SF) [44]	General satisfaction with life and concerns over social and vocational issues related to diabetes	2006	18	Disease impact Disease-related worries	5-point Likert scale	Diabetes specific	*Self-report* 10–18 years

DISABKIDS [49]	Health-related quality of life of children and adolescents with chronic medical conditions	2006	37	Mental Independence Emotion Social Inclusion Exclusion Physical Physical limitation Medical treatment	5-point Likert scale	Chronic-generic	*Self-report* 4–7 years (6-item version) 8–16 years *Proxy* 8–16 years 4–7 years (6-item version)

DISABKIDS, chronic generic module, short form (DCGM-12) [50]	General subjective chronic generic quality of life	2006	12	Mental Independence Emotion Social Inclusion Exclusion Physical Physical limitation Medical treatment	5-point Likert scale	Chronic-generic	*Self-report* 4–7 years (6-item version) 8–16 years *Proxy* 8–16 years 4–7 years (6-item version)

DISABKIDS, disease-specific module for diabetes (DM) [22]	Diabetes- specific quality of life	2006	13	Impact (acceptance) Treatment	5-point Likert scale	Diabetes specific	*Self-report* 8–16 years *Proxy* 8–16 years

KINDL questionnaire [59]	Health-related quality of life	1994	24 + 6 item “disease module”	Psychological well-being Social relationships Physical function Everyday life activities	5-point Likert scale	Generic	*Self-report* 4–6 years (interview based) 7–13 years 14–17 years *Proxy* 3–6 years 7–17 years

## Data Availability

The present mapping review is registered with the Research Registry and the unique identifying number is: researchregistry890. The review protocol can be accessed online at Research Registry [79]. Louise Norman Jespersen, Tue Helms Andersen, and Dan Grabowski conducted the systematic mapping review since the time of registration of the protocol, and the list of authors has therefore been updated. Data extraction for all included articles is available at: https://gapmap.danishdiabetesknowledgecenter.dk/qolchildren.

## Additional Points

A strength of our study is the extensive review of the literature and the systematic identification and presentation of QoL instruments used for children with diabetes. To be able to fully guide the readers in selection of measures, we are aware that a necessary next step is to conduct a quality assessment of a selection of the included studies. This is beyond the scope of this current paper, but will be initiated in the immediate future.

## Supplementary Materials

Supplementary Materials Supplementary 1: search documentation. Supplementary 2: excluded articles.
