# Supplementary material for: Comprehensive Overview of Quality of Life Instruments Used in Studies of Children with Diabetes: A Systematic Mapping Review
Source: Pediatr Diabetes. 2023 Sep 25;2023:8885973. doi: 10.1155/2023/8885973 (PMC12017037; doi:10.1155/2023/8885973)
Supplement: Supplementary Materials — Supplementary 1: search documentation. Supplementary 2: excluded articles. [file 8885973.f1.docx]

# **Supplementary Material 1: Search documentation**

## *Key concepts in the search strategy*

Concept 1: Diabetes. The concept ‘Diabetes’ was searched for in two parts:

1. Relevant subject headings, e.g. the Medical Subject Heading (MeSH) *Diabetes Mellitus.*
2. Free-text search in the title, abstract and keyword fields. We searched for the term *diabet* wich was truncated to include all forms of diabetes.

Concept 1: Children. The concept ‘Children and adolescents’ was searched for in two parts:

1. Relevant subject headings, e.g. the Medical Subject Heading (MeSH) *Child.*
2. Free-text search in the title, abstract and keyword fields. We searched for multiple synonyms including *child*, *adolescent*, *teen* etc. All was truncated to include different word endings.

Concept 3: Quality of life. The concept ‘Quality of life’ was searched for in two parts:

1. Relevant subject headings, e.g. the Medical Subject Heading (MeSH) *Quality of Life.*
2. Free-text search in the title, abstract and keyword fields. We searched for multiple synonyms and acronyms including *life quality, qol, hrqol* etc. All was truncated to include different word endings.

*Information sources and methods*

Initially, we searched the databases MEDLINE, Embase, PsycInfo, CINAHL and ERIC from inception to 13 February 2020. An updated search in all five databases was conducted on 18 January 2022.

*Contact with authors*

If no full-text report was available online or it could not be obtained from the Danish Royal Library, an e-mail was sent to the first or corresponding author of the report. The author was then given one month to answer and if no answer was received the report was excluded.

*Searches and results*

All complete search strategies with results and comments are listed in table 1 and 2. This includes the added date limit used for the updated search. Because the Danish Royal Library changed its access to the databases PsycInfo and ERIC, the initial search was done in the EBSCO interface and the updated search in the Ovid and ProQuest interfaces respectively.

## *Table 1: Searches in the databases MEDLINE and Embase*

| **#** | **[MEDLINE (Ovid)]** | | | **#** | **[Embase (Ovid)]** | | | **Comments** |
| --- | --- | --- | --- | --- | --- | --- | --- | --- |
| *Diabetes* | | | | | | | | |
| 1 | exp Diabetes Mellitus/ | 417334 | | 1 | exp diabetes mellitus/ | 926727 | | Subject headings for the population of interest (people with diabetes). |
| 2 | diabet$.ti,ab,kf,kw. | | 619100 | 2 | diabet$.ti,ab,kw. | | 916790 | Free-text term for the population of interest (people with diabetes). |
| 3 | 1 or 2 | | 676499 | 3 | 1 or 2 | | 1099843 |  |
| *Children and adolescents* | | | | | | | | |
| 4 | exp Child/ | | 1879153 | 4 | child/ or abandoned child/ or adopted child/ or boy/ or brain damaged child/ or "child of impaired parents"/ or foster child/ or gifted child/ or girl/ or handicapped child/ or hospitalized child/ or institutionalized child/ or orphaned child/ or preschool child/ or school child/ or single parent child/ or toddler/ or unwanted child/ | | 1952658 | Subject headings for the population of interest (children). |
| 5 | exp Adolescent/ | | 1991965 | 5 | exp adolescent/ | | 1483914 | Subject headings for the population of interest (adolescents). |
| 6 | exp Pediatrics/ | | 56999 | 6 | exp pediatrics/ | | 103722 | Subject headings for the population of interest (pediatrics). |
| 7 | (adolescen$ or child$ or pediatric$ or preadolescen$ or preteen$ or preschool$ or pre-school$ or teen$ or tween$ or youth$ or toddler$).ti,ab,kf,kw. | | 1728911 | 7 | (adolescen$ or child$ or pediatric$ or preadolescen$ or preteen$ or preschool$ or pre-school$ or teen$ or tween$ or youth$ or toddler$).ti,ab,kw. | | 2128273 | Free-text terms for the population of interest (Children and adolescents). The search line has synonyms for the concept of interest. |
| 8 | or/4-7 | | 3514290 | 8 | or/4-7 | | 3390813 |  |
| *Quality of life* | | | | | | | | |
| 9 | Quality of Life/ | | 188266 | 9 | exp quality of life/ | | 474372 | Subject headings for the outcome of interest (quality of life). |
| 10 | (life quality$ or hrqol$ or qol$ or quality of life$).ti,ab,kf,kw. | | 276769 | 10 | (life quality$ or hrqol$ or qol$ or quality of life$).ti,ab,kw. | | 448901 | Free-text terms for the outcome of interest (quality of life). The search line has synonyms for the concept of interest. |
| 11 | 9 or 10 | | 332291 | 11 | 9 or 10 | | 580900 |  |
| 12 | 3 and 8 and 11 | | 2473 | 12 | 3 and 8 and 11 | | 4178 | Combining key concepts of interest. |
| *Animal filter* | | | | | | | | |
| 13 | exp Animals/ not Humans/ | | 4673892 | 13 | (exp animal/ or nonhuman/) not exp human/ | | 6289504 | Excluding animal studies from the search result. |
| 14 | 12 not 13 | | 2472 | 14 | 12 not 13 | | 4161 |  |
| *Exclude medline journal limit* | | | | | | | | |
|  |  | |  | 15 | limit 14 to exclude medline journals | | 437 | Excluding MEDLINE journals from the search result in Embase. |
| Updating search | | | | | | | | |
| 15 | limit 14 to (dt="20200213-20251231" or ez="20200213-20251231") | | 350 | 16 | limit 15 to (dc="20200213-20251231" or rd="20200213-20251231") | | 210 | Updating search using date filters. |

## *Table 2: Searches in the databases PsycInfo, CINAHL and ERIC*

| **#** | **[PsycInfo (EBSCO)]** | | | **#** | **[CINAHL (EBSCO)]** | | | **#** | **[ERIC (EBSCO)]** | | | | **Comments** |
| --- | --- | --- | --- | --- | --- | --- | --- | --- | --- | --- | --- | --- | --- |
| *Diabetes* | | | | | | | | | | | | | |
| 1 | DE "Diabetes" OR DE "Diabetes Mellitus" OR DE "Type 2 Diabetes" | 17,589 | | 1 | (MH "Diabetes Mellitus+") | 147,868 | | 1 | DE "Diabetes" | 923 | | | Subject headings for the population of interest (people with diabetes). |
| 2 | TI (diabet*) OR AB (diabet*) OR KW (diabet*) | | 30,630 | 2 | TI (diabet*) OR AB (diabet*) | | 176,184 | 2 | TI (diabet*) OR AB (diabet*) OR KW (diabet*) | | | 1,272 | Free-text term for the population of interest (people with diabetes). |
| 3 | S1 OR S2 | | 31,017 | 3 | S1 OR S2 | | 208,876 | 3 | S1 OR S2 | | | 1,349 |  |
| *Children and adolescents* | | | | | | | | | | | | | |
|  |  | |  | 4 | (MH "Child") OR (MH "Child, Abandoned") OR (MH "Child, Adopted") OR (MH "Child, Disabled") OR (MH "Child, Foster") OR (MH "Child, Gifted") OR (MH "Child, Hospitalized") OR (MH "Child, Institutionalized") OR (MH "Child, Medically Fragile") OR (MH "Child, Preschool") OR (MH "Latchkey Children") OR (MH "Only Child") | | 475,462 | 4 | DE "African American Children" OR DE "Grandchildren" OR DE "Hospitalized Children" OR DE "Latchkey Children" OR DE "Migrant Children" OR DE "Minority Group Children" OR DE "Preadolescents" OR DE "Young Children" OR DE "Children" OR DE "Preschool Children" OR DE "Toddlers" | | | 101,236 | Subject headings for the population of interest (children). |
|  |  | |  | 5 | (MH "Adolescence+") | | 480,139 | 5 | DE "Adolescents" | | | 50,238 | Subject headings for the population of interest (adolescents). |
| 4 | DE "Pediatrics" | | 25,930 | 6 | (MH "Pediatric Nurse Practitioners") OR (MH "Pediatric Surgery") OR (MH "Pediatric Cardiology") OR (MH "Rehabilitation, Pediatric") OR (MH "Association of Pediatric Oncology Nurses") OR (MH "National Association of Pediatric Nurse Associates and Practitioners") OR (MH "Pediatric Advanced Life Support") OR (MH "Pediatric Oncology Nursing") OR (MH "Society of Pediatric Nurses") OR (MH "Childhood Neoplasms") OR (MH "Pediatric Units+") OR (MH "Pediatric Occupational Therapy") OR (MH "Hospitals, Pediatric") OR (MH "Pediatric Physical Therapy") | | 38,846 | 6 | DE "Pediatrics" | | | 1,103 | Subject headings for the population of interest (pediatrics). |
| 5 | TI (adolescen* or child* or pediatric* or preadolescen* or preteen* or preschool* or pre-school* or teen* or tween* or youth* or toddler*) OR AB (adolescen* or child* or pediatric* or preadolescen* or preteen* or preschool* or pre-school* or teen* or tween* or youth* or toddler*) OR KW (adolescen* or child* or pediatric* or preadolescen* or preteen* or preschool* or pre-school* or teen* or tween* or youth* or toddler*) | | 890,184 | 7 | TI (adolescen* or child* or pediatric* or preadolescen* or preteen* or preschool* or pre-school* or teen* or tween* or youth* or toddler*) OR AB (adolescen* or child* or pediatric* or preadolescen* or preteen* or preschool* or pre-school* or teen* or tween* or youth* or toddler*) | | 583,730 | 7 | TI (adolescen* or child* or pediatric* or preadolescen* or preteen* or preschool* or pre-school* or teen* or tween* or youth* or toddler*) OR AB (adolescen* or child* or pediatric* or preadolescen* or preteen* or preschool* or pre-school* or teen* or tween* or youth* or toddler*) OR KW (adolescen* or child* or pediatric* or preadolescen* or preteen* or preschool* or pre-school* or teen* or tween* or youth* or toddler*) | | | 368,875 | Free-text terms for the population of interest (Children and adolescents). The search line has synonyms for the concept of interest. |
| 6 | S4 OR S5 | | 890,923 | 8 | S4 OR S5 OR S6 OR S7 | | 965,632 | 8 | S4 OR S5 OR S6 OR S7 | | | 383,236 |  |
| *Quality of life* | | | | | | | | | | | | | |
| 7 | DE "Quality of Life Measures" OR DE "Quality of Life" OR DE "Health Related Quality of Life" | | 55,070 | 9 | (MH "Quality of Life") | | 101,996 | 9 | DE "Quality of Life" | | 6,161 | | Subject headings for the outcome of interest (quality of life). |
| 8 | TI (life quality* or hrqol* or qol* or quality of life*) OR AB (life quality* or hrqol* or qol* or quality of life*) OR KW (life quality* or hrqol* or qol* or quality of life*) | | 74,928 | 10 | TI (life quality* or hrqol* or qol* or quality of life*) OR AB (life quality* or hrqol* or qol* or quality of life*) | | 116,206 | 10 | TI (life quality* or hrqol* or qol* or quality of life*) OR AB (life quality* or hrqol* or qol* or quality of life*) OR KW (life quality* or hrqol* or qol* or quality of life*) | | 6,792 | | Free-text terms for the outcome of interest (quality of life). The search line has synonyms for the concept of interest. |
| 9 | S7 OR S8 | | 85,710 | 11 | S9 OR S10 | | 154,654 | 11 | S9 OR S10 | | 9,722 | |  |
| 10 | S3 AND S6 AND S9 | | 392 | 12 | S3 AND S8 AND S11 (including a filter to exclude MEDLINE journals) | | 407 | 12 | S3 AND S8 AND S11 | | 16 | | Combining key concepts of interest. |
| Updating search | | | | | | | | | | | | | |
| 11 | limit 10 to up="20200213-20251231" (update of search conducted in Ovid interface) | | 36 | 13 | EM 20200213-20251231 | | 723,389 | 13 | Applied filters 2020-2029 (Update of search conducted in ProQuest) | | 1 | | Updating search using date filters. |
|  |  | |  | 14 | S12 AND S13 | | 125 |  |  | |  | |  |

# **Supplementary Material 2: Excluded articles**

## *Articles in other languages than English, Danish, Norwegian and Swedish*

| Afshar M, Memarian R and Mohammadi I ;. (2014). Investigating the relationship between quality of life, self-care capability and HbA1c level in diabetic adolescents.. *Feyz Journal of Kashan University of Medical Sciences*, 18(1), pp.68-75. |
| --- |
| Alavi A, Parvin N and Salehian T ; Samipoor V ;. (2010). Assessment of the quality of life of children and adolescents with type 1 diabetes: Child and parental views. *Scientific Journal of Kurdistan University of Medical Sciences*, 15(1), pp.46-52. |
| Bartus B. (1998). [Psychosocial situation and quality of life of children and adolescents with diabetes mellitus].. *Psychosoziale Situation und Lebensqualitat von Kindern und Jugendlichen mit Diabetes mellitus.*, 17(4), pp.155-7. |
| Çakin Memik, Nursu and Ağaoğlu Belma ; Coşkun Ayşen ; Hatun Şükrü ; Ayaz Mohammad ; Karakaya Işik ;. (2007). Tip 1 diyabetes mellitusu olan çocuk ve ergenlerin yaşam kalitesi algilarinin dğerlendirilmesi = Evaluation of quality of life in children and adolescents with type 1 diabetes mellitus. *Çocuk ve Gençlik Ruh Sağliği Dergisi*, 14(3), pp.133-138. |
| Duras E, Bezen D and Ozkaya O ; Dursun H ;. (2018). Evaluation of the quality of life of patients followed up with diagnosis of type 1 diabetes mellitus. *Tip 1 diyabetes mellitus tanisi ile izlenmekte olan hastalarin yasam kalitesi duzeylerinin degerlendirilmesi*, 16(2), pp.72-85. |
| Garcia Leila F Dos S and Manna Thais Della; Passone Caroline de Gouveia Buff; Oliveira Lygia Spassapan de;. (2018). Translation and validation of Pediatric Quality of Life InventoryTM 3.0 Diabetes Module (PedsQLTM 3.0 Diabetes Module) in Brazil-Portuguese language.. *Jornal de pediatria*, 94(6), pp.680-688. |
| Geirhos Agnes, Domhardt Matthias and Baumeister Harald ; Galler Angela ; Reinauer Christina ; Warschburger Petra ; Muller-Stierlin Annabel S; Minden Kirsten ; Temming Svenja ; Holl Reinhard W;. (2020). Mental comorbidity in adolescents and young adults with type 1 diabetes. *Psychische Komorbiditaten bei Jugendlichen und jungen Erwachsenen mit Typ-1-Diabetes*, 15(6), pp.487-497. |
| Graue Marit, Wentzel-Larsen Tore and Bru Edvin ; Hanestad Berit Rokne; Sovik Oddmund ;. (2004). The coping styles of adolescents with type 1 diabetes are associated with degree of metabolic control.. *Diabetes care*, 27(6), pp.1313-7. |
| Kermansaravi F, Navidian A and Ansarymoghadam A ;. (2012). Quality of life in type 1 diabetic adolescents in Zahedan (2011). *Iranian Journal of Endocrinology and Metabolism*, 13(6), pp.. |
| Lange K, Kleine T and Danne T ; AG Diabetesschulung fur Eltern;. (2011). [Initial education for parents of children with diabetes: effort and outcomes in children and parents].. *Initialschulung fur Eltern von Kindern mit Diabetes: Aufwand und Effekte bei Kindern und Eltern.*, 136(21), pp.1106-10. |
| Laptev D N and Peterkova V A;. (2017). Use of telemedicine improves glycemic control and quality of life in type 1 diabetes children on insulin pump therapy. *Diabetes Mellitus*, 20(6), pp.420-426. |
| Laptev Dmitry N and Emelyanov Andrey O; Samoilova Yulia G; Khramova Elena B; Petriaikina Elena E; Rybkina Irina G; Filimonova Alla Yu; Peterkova Valentina A;. (2020). [Remote monitoring and treatment of children and adolescents with type 1 diabetes].. *Problemy endokrinologii*, 66(4), pp.50-60. |
| Li Rong, Xiong Feng and Hu Yujuan ; Cheng Xinran ; Luo Yanhong ; Liang Qinxiang ; Wang Li ; Luo Shunqing ; Zhu Min ;. (2013). [Survey on quality of life in children and adolescents with type 1 diabetes].. *Zhejiang da xue xue bao. Yi xue ban = Journal of Zhejiang University. Medical sciences*, 42(4), pp.388-95. |
| Lichtenberger-Geslin L, Boudailliez B and Braun K ; Bach V ; Mercier A ; Bony-Trifunovic H ;. (2013). [Does insulin pump therapy improve quality of life and satisfaction in children and adolescents with type 1 diabetes?].. *La pompe a insuline ameliore-t-elle la qualite de vie et la satisfaction des enfants et adolescents diabetiques de type 1 ?*, 20(3), pp.248-56. |
| Miranda Velasco, M J and Dominguez Martin ; E ; Arroyo Diez ; F J ; Mendez Perez ; P ; Gonzalez de Buitrago Amigo; J ;. (2012). [Health related quality of life in type 1 diabetes mellitus].. *Calidad de vida relacionada con la salud en la diabetes mellitus tipo 1.*, 77(5), pp.329-33. |
| Monje M J.A and Almagia E B;. (2008). Self-efficacy, social support and quality of life of adolescents with chronic illnesses. *Autoeficacia and apoyo social y calidad de vida en adolescentes con enfermedades cronicas*, 26(2), pp.165-172. |
| Nahid Hemmat Makan, Fatemeh Golshani and Anita Baghdasarians ; Sozan Emamipour ;. (2021). Effect of Self-Care Education on Blood Glucose, Diabetic Quality of Life, and Emotional Behavioral Disorders in Adolescents with Diabetes.. *Journal of Diabetic Nursing*, 9(1), pp.1274-1286. |
| Otto T, Stralka R and Haupt A ; Bruns K ; Danne T ;. (2014). Insulin therapy in children and adolescents with type 1 diabetes: A survey of preferences in affected individuals and their families. *Insulintherapie bei Kindern und Jugendlichen mit Typ-1-Diabetes: Welche Praferenzen haben die Betroffenen?*, 19(3), pp.126-136. |
| Przybyszewski B, Czerwionka-Szaflarska M and Zbikowska-Bojko M ;. (2008). Quality of life in children and youth with type 1 diabetes receiving continuous subcutaneous insulin infusion. *Analiza jakosci zycia dzieci i mlodziezy chorych na cukrzyce typu 1 leczonych za pomoca ciaglego podskornego wlewu insuliny*, 83(1), pp.54-62. |
| Lima Lívia AP and Weffort Virgínia RS; Borges Maria de Fátima;. ( ). Quality of life evaluation of children with type 1 Diabetes mellitus.. *Ciencia and Cuidado e Saude*, 10(1), pp.127-133. |
| Safarabadi-Farahani T, Ali-Akbar M and Safarabadi-Farahani A ; Haghani H ;. ( ). Quality of life in young people with type 1 diabetes in relation to age and gender.. *Iran Journal of Nursing*, 23(68), pp.1p-1p. |
| Almeida J Paulo and Pereira M Graça;. ( ). Questionário de avalição da qualidade de vida para adolescentes com diabetes tipo 1: Estudo de validaçãao do DQOL = Adaptation of a questionnaire on quality of life for adolescents with Type 1 diabetes. *Análise Psicológica*, 26(2), pp.295-305. |
| Rodriguez Perez, C and Lizondo Escuder ; A ; Lopez Garcia ; M J ; Escriva Cholbi ; L ; Alpera Lacruz ; R ; Collado Perez ; C ;. (2008). [A study of variability in glycaemia in children and adolescents with diabetes mellitus type 1 on treatment with insulin glargine].. *Estudio de la variabilidad glucemica en ninos y adolescentes con diabetes tipo 1 en tratamiento con insulina glargina.*, 69(5), pp.426-31. |
| Sargazi Shad T, Kermansaravi F and Navidian A ;. (2018). Effect of the family-centered empowerment model on quality of life and self-efficacy in adolescents with type 1 diabetes referring to the Ali Asghar's clinic in Zahedan, 2016. *Iranian Journal of Endocrinology and Metabolism*, 19(5), pp.330-339. |
| Schiel R, Kaps A and Weihs-Godenrath T ; Kostin S ;. (2012). A structured treatment and teaching concept focussed on pedagogical skills for children and adolescents with type 1 diabetes mellitus. *Ein erlebnispadagogisch fokussiertes strukturiertes Behandlungs- und Schulungskonzept fur Kinder und Jugendliche mit Typ-1-Diabetes mellitus*, 7(3), pp.193-197. |
| Schiel R, Burgard D and Perenthaler T J; Steveling A ;. (2017). What is the effect of an in-patient rehabilitation for children and adolescents with type 1 diabetes mellitus - An analysis of 901 patients over a period of 6 years. *Was bewirkt eine Rehabilitation bei Kindern und Jugendlichen mit Typ-1-Diabetes mellitus - Analyse von 901 Patienten uber einen Zeitraum von 6 Jahren*, 12(2), pp.127-134. |
| Schonle E J. (1987). [Intensive insulin therapy in adolescents with type 1 diabetes mellitus: initial experiences with a semiautomatic insulin injection device (the insulin pen)].. *Intensivierte Insulin-Therapie bei Adoleszenten mit Diabetes mellitus Typ I: Erste Erfahrungen mit dem halbautomatischen Insulin-Injektionsgerat (Insulin-Pen).*, 117(45), pp.1756-60. |
| Sendela Joanna, Zduńczyk Beata and Trippenbach-Dulska Hanna ; Szypowska Agnieszka ;. (2015). Występowanie objawów depresji u dzieci w wieku szkolnym z cukrzycą typu 1—Badanie kwestionariuszowe = Prevalence of depressive symptoms in school-aged children with type 1 diabetes—A questionnaire study. *Psychiatria Polska*, 49(5), pp.1005-1016. |
| Stadler Marietta, Zlamal-Fortunat Sandra and Schutz-Fuhrmann Ingrid ; Rami-Merhar Birgit ; Schober Edith ; Kautzky-Willer Alexandra ; Weitgasser Raimund ; Prager Rudolf ; Bischof Martin ;. (2012). [Guidelines for insulin pump therapy in children and adults].. *Leitlinien Insulinpumpentherapie bei Kindern und Erwachsenen (Fur den Ausschuss Insulinpumpentherapie der Osterreichischen Diabetesgesellschaft).*, 124 Suppl 2, pp.123-8. |
| Stahl-Pehe A, Castillo Reinado K and Bachle C ; Lange K ; Holl R W; Rosenbauer J ;. (2014). Quality of life in youths with early-onset type 1 diabetes. Disagreement between self- and parent-reports. *Lebensqualitat von jugendlichen mit fruhmanifestem diabetes mellitus typ 1. Unterschiede im selbst- und elternurteil*, 82(3), pp.417-429. |
| Weyhreter H, Holl R W and Beerstecher A M; Borsch M ;. (2008). [Additional treatment supporting standard care for children and adolescents with diabetes mellitus type I - indication, acceptance and outcome: results from a multi-centre observational study].. *Interventionen zur Unterstutzung der Regelversorgung von Kindern und Jugendlichen mit Diabetes Typ I - Indikationen and Inanspruchnahme und Effekte: Ergebnisse einer multizentrischen Beobachtungsstudie.*, 220(2), pp.70-6. |
| Wiehe Katharina. (2006). [Stroke of fate or personal challenge--subjective theories of illness as risk or protective factors in coping with chronic pediatric illness].. *Zwischen Schicksalsschlag und Lebensaufgabe-- Subjektive Krankheitstheorien als Risiko- oder Schutzfaktoren der Bewaltigung chronischer Krankheit im Kindesalter.*, 55(1), pp.3-22. |
| Wiehe Katharina. (2006). Zwischen Schicksalsschlag und Lebensaufgabe--Subjektive Krankheitstheorien als Risiko- oder Schutzfaktoren der Bewältigung chronischer Krankheit im Kindesalter = Stroke of fate or personal challenge--Subjective theories of illness as risk or protective factors in coping with chronic pediatric illness. *Praxis der Kinderpsychologie und Kinderpsychiatrie*, 55(1), pp.3-22. |
| Wiss M, Perrot A and Sauvage D ;. (2000). [Psychological aspects of insulin-dependent diabetes].. *Les aspects psychologiques du diabete insulino-dependant.*, (196), pp.18-21. |
| Wurst E, Herle M and Fuiko R ; Hajszan M ; Katkhouda C ; Kieboom A ; Schubert M T;. (2002). [The quality of life of chronically ill and psychiatrically disturbed children. Initial experiences with an inventory for assessing quality of life in children and adolescents].. *Zur Lebensqualitat chronisch kranker und psychisch auffalliger Kinder. Erste Erfahrungen mit dem Inventar zur Erfassung der Lebensqualitat bei Kindern und Jugendlichen (ILK).*, 30(1), pp.21-8. |
| Yildiz Akkus, Sevilay and Butun Ayhan ; Aynur ;. (2020). Investigation of the behavioral problems and life quality of the children with chronic diseases. *Kronik hastaligi olan cocuklarin davranislarinin ve yasam kalitelerinin Incelenmesi*, 14(2), pp.129-135. |
| Yurteri N, Pekcanlar Akay A and Ellidokuz H ;. (2019). Assessment of health-related quality of life in children with ADHD by comparison with type 1 diabetes and healthy control groups. *DEHB'li cocuklarda saglikla ilgili yasam kalitesinin tip 1 diyabet ve saglikli kontrol gruplariyla karsilastirilarak degerlendirilmesi*, 20(5), pp.539-547. |

*Articles not able to retrieve*

| Bujang M A, Supramaniam P and Haniff J ; Muninathan P ; Jalaludin M Y; Zain F M; Hua J H.Y;. (2013). The association between self-care practices, HbA1c and quality of life for type 1 DM in children and adolescents. *International Medical Journal*, 20(3), pp.317-319. |
| --- |
| Dumontet Fabrice and Mochon Stephanie . (2012). [Insulinotherapy education for children].. *L'education a l'insulinotherapie fonctionnelle chez l'enfant.*, (769), pp.19-24. |
| Glowinska-Olszewska Barbara, Jablonska Jolanta and Otocka Agnieszka ; Florys Bozena ; Jamiolkowska Milena ; Tobiaszewska Monika ; Semeran Kornel ; Peczynska Jadwiga ; Luczynski Wlodzimierz ; Bossowski Artur ;. (2011). [Usefulness of short-term usage of real time continuous glucose monitoring system in achieving metabolic control improvement in adolescents with type 1 diabetes].. *Przydatnosc krotkoterminowego wykorzystania systemu ciaglego monitorowania glikemii w czasie rzeczywistym w uzyskaniu poprawy metabolicznej u nastolatkow z cukrzyca typu 1.*, 17(4), pp.179-87. |
| Goldbeck L, Braun J and Storck M ; Tonnessen D ; Weyhreter H ; Debatin K M;. (2001). [Adaptation of parents to the diagnosis of a chronic disease in their child].. *Adaptation von Eltern an eine chronische Erkrankung ihres Kindes nach der Diagnosestellung.*, 51(2), pp.62-7. |
| Hirose Masakazu and Kawamura Tomoyuki . (2016). [Diabetes education for patients and their surroundings in pediatrics].. *Nihon rinsho. Japanese journal of clinical medicine*, 74 Suppl 2, pp.512-6. |
| Hoey Hilary M C V. (2003). Background and methods for evaluating quality of life in children and adolescents with diabetes.. *Acta bio-medica : Atenei Parmensis*, 74 Suppl 1, pp.7-12. |
| Matam P, Kumaraiah V and Munichoodappa C ; Kumar K M; Aravind S ;. (2000). Behavioural intervention in the management of compliance in young type-I diabetics.. *The Journal of the Association of Physicians of India*, 48(10), pp.967-71. |
| Frey MA. ( ). Measurement corner. Health-related quality of life: promises and pitfalls.. *Journal of Child & Family Nursing*, 4(1), pp.63-67. |
| Novato Tatiana de Sa and Grossi Sonia Aurora Alves; Kimura Miako. (2007). [Quality of Life Instrument for Youths with Diabetes].. *Instrumento de Qualidade de Vida para Jovens com Diabetes (IQVJD).*, 28(4), pp.512-9. |
| Pirard P. (2013). Type 1 diabetes and sailing... a perfect marriage?. *Diabete de type i et voile sportive. Un mariage parfait ?*, 132(3), pp.117-120. |
| R C. (2015). Benefits of Insulin Pumps.. *School Health Alert*, 30(5), pp.3-3. |
| Rendeli C, Padua L and Ausili E ; Crea F ; Pedace C ; Caliandro P ; Selvaggio E ; Marietti G ;. (2003). Quality of life in young diabetic patients.. *Diabetes and nutrition & metabolism*, 16(4), pp.251-6. |
| Schober E, Borkenstein M and Frisch H ;. (1987). [Basic-bolus therapy of diabetic children and adolescents using Novo Pens].. *Basis-Bolus-Therapie bei diabetischen Kindern und Jugendlichen unter Verwendung des Novo Pens.*, 99(9), pp.312-3. |
| Shilbayeh Sireen A and Alhussain Wedyan A; Alhibs Alanoud S; Almalki Najla G; Almajhad Nouf A; Nouri Samar T; Alsaleh Sarah M;. (2021). Type 2 diabetes mellitus and its effect on quality of life among adolescent population: A retrospective cohort study in Saudi Arabia. *International Journal of Pharmaceutical Research*, 13(1), pp.2133-2144. |
| Tzischinsky O, Tenenbaum-Rakover Y and Shapira S ; Rabin L ; Hess O ; Haimov I ;. ( ). Sleep quality, quality of life, and cognitive performance among adolescents with type 1 diabetes.. *Sleep Medicine*, 64, pp.S394-S394. |
| Peng X, Cui Y and Dong L ;. ( ). Study on relativity among coping styles, blood glucose control and quality of life of children and adolescent with diabetes mellitus.. *Chinese Nursing Research*, 24(6A), pp.1421-1423. |
| Suba I, Halmos T and Litvai G ;. (1979). [The significance of disease in the life of the patient in juvenile type (insulin deficient) diabetes mellitus].. *A betegseg jelentosege az egyen eleteben juvenilis tipusu (inzulinhianyos) diabetes mellitusban.*, 120(49), pp.2989-93. |

*Articles excluded after full text screening because no quantitative measure was used*

| AlAhmed Ohoud, Sivaraman Vidya and Moore-Clingenpeel Melissa ; Ardoin Stacy P; Bout-Tabaku Sharon ; CARRA registry investigators;. (2020). Autoimmune thyroid diseases, autoimmune hepatitis, celiac disease and type 1 diabetes mellitus in pediatric systemic lupus erythematosus: Results from the CARRA Legacy Registry.. *Lupus*, 29(14), pp.1926-1936. |
| --- |
| Almeida Andre Coelho, Batista Rita and Correia Filipa ; Candido Cristina ; Martins Vania ; Barros Marta ;. (2021). Hospital admissions in patients with type 1 diabetes mellitus, before and after 2011. *13th Excellence in Pediatrics Conference and EiP 2021. Virtual.*, 8, pp.. |
| AlMutairi Faisal F J and Pani Sharat C; Alrobaie Fahd M; Ingle Navin A;. (2020). Relationship between type-I diabetes mellitus and oral health status and oral health-related quality of life among children of Saudi Arabia.. *Journal of family medicine and primary care*, 9(2), pp.647-651. |
| Anderson Jeffrey B and Czosek Richard J; Knilans Timothy K; Marino Bradley S;. (2012). The effect of paediatric syncope on health-related quality of life.. *Cardiology in the young*, 22(5), pp.583-8. |
| Awadalla Abdel W and Ohaeri Jude U; Tawfiq Adel M; Al-Awadi Shafika A;. (2006). Subjective quality of life of outpatients with diabetes: comparison with family caregivers' impressions and control group.. *Journal of the National Medical Association*, 98(5), pp.737-45. |
| Barakat Caroline, Yousufzai Susan Jamuria and Booth Alison ; Benova Lenka ;. (2021). Prevalence of and risk factors for diabetes mellitus in the school-attending adolescent population of the United Arab Emirates: a large cross-sectional study.. *BMJ open*, 11(9), pp.e046956. |
| Burgmann Johanna, Biester Torben and Grothaus Julia ; Kordonouri Olga ; Ott Hagen ;. (2020). Pediatric diabetes and skin disease (PeDiSkin): A cross-sectional study in 369 children, adolescents and young adults with type 1 diabetes.. *Pediatric diabetes*, 21(8), pp.1556-1565. |
| Carroll Aaron E, DiMeglio Linda A; Stein Stephanie and Marrero David G;. (2011). Using a cell phone-based glucose monitoring system for adolescent diabetes management.. *The Diabetes educator*, 37(1), pp.59-66. |
| Christie Deborah, Thompson Rebecca and Sawtell Mary ; Allen Elizabeth ; Cairns John ; Smith Felicity ; Jamieson Elizabeth ; Hargreaves Katrina ; Ingold Anne ; Brooks Lucy ; Wiggins Meg ; Oliver Sandy ; Jones Rebecca ; Elbourne Diana ; Santos Andreia ; Wong Ian C K; O'Neill Simon ; Strange Vicki ; Hindmarsh Peter ; Annan  Francesca ; Viner Russell ;. (2014). Structured, intensive education maximising engagement, motivation and long-term change for children and young people with diabetes: a cluster randomised controlled trial with integral process and economic evaluation - the CASCADE study.. *Health technology assessment (Winchester and England)*, 18(20), pp.1-202. |
| Chuang Janet, Zeller Meg H and Inge Thomas ; Crimmins Nancy ;. (2013). Bariatric surgery for severe obesity in two adolescents with type 1 diabetes.. *Pediatrics*, 132(4), pp.e1031-4. |
| Chute Christopher G and Hart Lacey A; Alexander Alex K; Jensen Daniel W;. (2014). The Southeastern Minnesota Beacon Project for Community-driven Health Information Technology: Origins, Achievements, and Legacy.. *EGEMS (Washington and DC)*, 2(3), pp.1101. |
| Cobry Erin C, Hamburger Emily and Jaser Sarah S;. (2020). Impact of the Hybrid Closed-Loop System on Sleep and Quality of Life in Youth with Type 1 Diabetes and Their Parents.. *Diabetes technology & therapeutics*, 22(11), pp.794-800. |
| Cumba-Aviles Eduardo and Saez-Santiago Emily . (2016). Research Program on Type 1 Diabetes and Youth Depression in Puerto Rico.. *Revista Puertorriquena de psicologia*, 27(1), pp.44-60. |
| Dawes Adam. (2019). The use of insulin pumps in children and young people: Past, present and future.. *Journal of Diabetes Nursing*, 23(5), pp.1-4. |
| De Ridder , Francesca and den Brinker ; Marieke ; De Block ; Christophe ;. (2019). The road from intermittently scanned continuous glucose monitoring to hybrid closed-loop systems. Part B: results from randomized controlled trials.. *Therapeutic advances in endocrinology and metabolism*, 10, pp.2042018819871903. |
| de Wit , Maartje and Snoek Frank J;. (2009). The DAWN MIND Youth program.. *Pediatric diabetes*, 10 Suppl 13, pp.46-9. |
| Dixon Brenner, Peter Chase and H ; Burdick Jonathan ; Fiallo-Scharer Rosanna ; Walravens Philippe ; Klingensmith Georgeanna ; Rewers Marian ; Garg Satish K;. (2005). Use of insulin glargine in children under age 6 with type 1 diabetes.. *Pediatric diabetes*, 6(3), pp.150-4. |
| Dorchy H. (2000). Insulin regimens and insulin adjustments in diabetic children, adolescents and young adults: personal experience.. *Diabetes & metabolism*, 26(6), pp.500-7. |
| Duru N S, Civilibal M and Elevli M ;. (2016). Quality of Life and Psychological Screening in Children with Type 1 Diabetes and their Mothers.. *Experimental and clinical endocrinology & diabetes : official journal and German Society of Endocrinology [and] German Diabetes Association*, 124(2), pp.105-10. |
| Eilander Minke, de Wit and Maartje ; Rotteveel Joost ; Maas-van Schaaijk ; Nienke ; Roeleveld-Versteegh Angelique ; Snoek Frank ;. (2016). Implementation of quality of life monitoring in Dutch routine care of adolescents with type 1 diabetes: appreciated but difficult.. *Pediatric diabetes*, 17(2), pp.112-9. |
| Eilander Minke Ma, de Wit and Maartje ; Rotteveel Joost ; Aanstoot Henk Jan; Bakker-van Waarde ; Willie M ; Houdijk Euphemia Cam; Nuboer Roos ; Winterdijk Per ; Snoek Frank J;. (2017). Disturbed eating behaviors in adolescents with type 1 diabetes. How to screen for yellow flags in clinical practice?.. *Pediatric diabetes*, 18(5), pp.376-383. |
| Elheeny A A H. (2020). Determinants of oral-health related quality of life and overall quality of life among early adolescents with type-1 diabetes.. *Community dental health*, 37(3), pp.199-204. |
| Fogel Jennifer L and Raymond Jennifer K;. (2020). Implementing Telehealth in Pediatric Type 1 Diabetes Mellitus.. *Pediatric clinics of North America*, 67(4), pp.661-664. |
| Gavrylenko Yurii V and Laiko Andriy A; Melnikov Oleg F; Rudenko Lilia M; Osadcha Tatiana M;. (2020). Clinical and laboratory peculiarities of children's state with chronic tonsilitis on type 1 diabetes.. *Wiadomosci lekarskie (Warsaw and Poland : 1960)*, 73(7), pp.1465-1469. |
| Goldbeck L. (2001). Parental coping with the diagnosis of childhood cancer: gender effects, dissimilarity within couples, and quality of life.. *Psycho-oncology*, 10(4), pp.325-35. |
| Goldbeck L and Braun R . (2003). LQ-KID: A computer-assisted method to assess health-related quality of life of children and adolescents with chronic conditions. *LQ-KID: Ein computergestutztes verfahren zur erfassung der lebensqualitat chronisch kranker kinder und jugendlicher*, 15(3), pp.117-126. |
| Grey Margaret, Whittemore Robin and Liberti Lauren ; Delamater Alan ; Murphy Kathryn ; Faulkner Melissa S;. (2012). A comparison of two internet programs for adolescents with type 1 diabetes: design and methods.. *Contemporary clinical trials*, 33(4), pp.769-76. |
| Grudziaz-Sekowska Justyna, Zamarlik Monika and Sekowski Kuba ;. (2021). Assessment of Selected Aspects of the Quality of Life of Children with Type 1 Diabetes Mellitus in Poland.. *International journal of environmental research and public health*, 18(4), pp.. |
| Hahl J, Hamalainen H and Sintonen H ; Simell T ; Arinen S ; Simell O ;. (2002). Health-related quality of life in type 1 diabetes without or with symptoms of long-term complications.. *Quality of life research : an international journal of quality of life aspects of treatment and care and rehabilitation*, 11(5), pp.427-36. |
| Hamman Richard F, Bell Ronny A; Dabelea Dana and D'Agostino Ralph B Jr; Dolan Lawrence ; Imperatore Giuseppina ; Lawrence Jean M; Linder Barbara ; Marcovina Santica M; Mayer-Davis Elizabeth J; Pihoker Catherine ; Rodriguez Beatriz L; Saydah Sharon ; SEARCH for Diabetes in Youth Study Group;. (2014). The SEARCH for Diabetes in Youth study: rationale, findings, and future directions.. *Diabetes care*, 37(12), pp.3336-44. |
| Haynes Emily, Ley Marissa and Talbot Pam ; Dunbar Margaret ; Cummings Elizabeth ;. (2021). Insulin Pump Therapy Improves Quality of Life of Young Patients With Type 1 Diabetes Enrolled in a Government-Funded Insulin Pump Program: A Qualitative Study.. *Canadian journal of diabetes*, 45(5), pp.395-402. |
| Herwig Jurgen, Scholl-Schilling Gabriele and Bohles Hans ;. (2007). Glycaemic control and hypoglycaemia in children, adolescents and young adults with unstable type 1 diabetes mellitus treated with insulin glargine or intermediate-acting insulin.. *Journal of pediatric endocrinology & metabolism : JPEM*, 20(4), pp.517-25. |
| Hoey Hilary and Hvidoere Study Group on Childhood Diabetes. (2009). Psychosocial factors are associated with metabolic control in adolescents: research from the Hvidoere Study Group on Childhood Diabetes.. *Pediatric diabetes*, 10 Suppl 13, pp.9-14. |
| Janzen Claude, Jennifer A and Hadjistavropoulos Heather D; Friesen Lindsay ;. (2014). Exploration of health anxiety among individuals with diabetes: prevalence and implications.. *Journal of health psychology*, 19(2), pp.312-22. |
| Kanner Sheri, Hamrin Vanya and Grey Margaret ;. (2003). Depression in adolescents with diabetes.. *Journal of child and adolescent psychiatric nursing : official publication of the Association of Child and Adolescent Psychiatric Nurses and Inc*, 16(1), pp.15-24. |
| Lane Wendy, Lambert Emma and George Jesso ; Rathor Naveen ; Thalange Nandu ;. (2021). Exploring the Burden of Mealtime Insulin Dosing in Adults and Children With Type 1 Diabetes.. *Clinical diabetes : a publication of the American Diabetes Association*, 39(4), pp.347-357. |
| Levy-Shraga Y, Elisha N and Ben-Ami M ; Boyko V ; Lerner-Geva L ; Ziv T ; Konvalina N ; Cohen O ; Pinhas-Hamiel O ;. (2016). Glycemic control and clinic attendance of emerging adults with type 1 diabetes at a transition care clinic.. *Acta diabetologica*, 53(1), pp.27-33. |
| Litton Jean, Rice Alan and Friedman Nancy ; Oden Jon ; Lee Mary M; Freemark Michael ;. (2002). Insulin pump therapy in toddlers and preschool children with type 1 diabetes mellitus.. *The Journal of pediatrics*, 141(4), pp.490-5. |
| Lugasi Tziona, Achille Marie and Blydt-Hansen Tom ; Clermont Marie-Jose ; Geoffroy Louis ; Legault Laurent ; Phan Veronique ; Bell Lorraine E;. (2013). Assessment of identity and quality of life in diabetic and renal transplant adolescents in comparison to healthy adolescents.. *Journal of clinical psychology in medical settings*, 20(3), pp.361-72. |
| Mahmud Farid H, Murray Joseph A; Kudva Yogish C; Zinsmeister Alan R; Dierkhising Ross A; Lahr Brian D; Dyck Peter J; Kyle Robert A; El-Youssef Mounif and Burgart Lawrence J; Van Dyke ; Carol T ; Brogan Deanna L; Melton L Joseph 3rd;. (2005). Celiac disease in type 1 diabetes mellitus in a North American community: prevalence, serologic screening, and clinical features.. *Mayo Clinic proceedings*, 80(11), pp.1429-34. |
| Mannucci E, Pala L and Rotella C M;. (2005). Long-term interactive group education for type 1 diabetic patients.. *Acta diabetologica*, 42(1), pp.1-6. |
| Marcovecchio et al. Adolescent type 1 Diabetes cardio-renal Intervention Trial Researc. (2009). Adolescent type 1 Diabetes Cardio-renal Intervention Trial (AdDIT).. *BMC pediatrics*, 9, pp.79. |
| Markowitz Jessica T and Antisdel Jeanne E; Butler Deborah A; Anderson Barbara J; Volkening Lisa K; Laffel Lori M. B;. (2010). Brief screening tool for disordered eating in diabetes.. *Diabetes Care*, 33(3), pp.495-500. |
| Meuleners Lynn B and Lee Andy H; Binns Colin W;. (2001). Assessing quality of life for adolescents in Western Australia. *Asia-Pacific Journal of Public Health*, 13(1), pp.40-44. |
| Muratalina Aigul, Smith-Palmer Jayne and Nurbekova Akmaral ; Abduakhassova Gulmira ; Zhubandykova Leila ; Roze Stephane ; Karamalis Manolis ; Shamshatova Gulzhakhan ; Demessinov Adi ; D'Agostino Nicola Dunne; Lynch Peter ; Yedigarova Larisa ; Klots Motty ; Valentine William ; Welsh John ; Kaufman Francine ;. (2015). Project Baiterek: A Patient Access Program to Improve Clinical Outcomes and Quality of Life in Children with Type 1 Diabetes in Kazakhstan.. *Value in health regional issues*, 7, pp.74-79. |
| Nakamura Nobue, Sasaki Nozomu and Kida Kaichi ; Matsuura Nobuo ;. (2009). Title: Health-related and diabetes-related quality of life (QOL) in Japanese children and adolescents with type 1 and type 2 diabetes.. *Pediatrics international : official journal of the Japan Pediatric Society*, , pp.. |
| Nguyen T Q and Vo T Q; Nguyen G H; Nguyen T D;. (2018). Assessment of health-related quality of life in patients with type II diabetes mellitus: A population-based study at a tertiary hospital. *Journal of Clinical and Diagnostic Research*, 12(6 Supplement 1), pp.LC44-LC51. |
| Noueiri Balsam and Nassif Nahla . (2018). Impact of Diabetes Mellitus Type 1 on Lebanese Families' Quality of Life.. *International journal of clinical pediatric dentistry*, 11(2), pp.61-65. |
| Petruzelkova Lenka, Jiranova Pavlina and Soupal Jan ; Kozak Milos ; Plachy Lukas ; Neuman Vit ; Pruhova Stepanka ; Obermannova Barbora ; Kolouskova Stanislava ; Sumnik Zdenek ;. (2021). Pre-school and school-aged children benefit from the switch from a sensor-augmented pump to an AndroidAPS hybrid closed loop: A retrospective analysis.. *Pediatric diabetes*, 22(4), pp.594-604. |
| Pierce Jessica S, Enlow Paul and Alderfer Melissa A; Wasserman Rachel ; Gurnurkar Shilpa ; O'Hara Erin ; Pendley Jennifer Shroff; Reed Michelle ; Welsh Kristine ; Brooks Kaley ; Taylor Alex ; Wysocki Tim ; Price Julia ;. (2021). Transdisciplinary Care for Adolescents With Type 1 Diabetes: Development of a Provider Cross-Discipline Training Curriculum.. *Diabetes Spectrum*, 34(4), pp.430-435. |
| Pinsker Jordan E, Muller Lars and Constantin Alexandra ; Leas Scott ; Manning Michelle ; McElwee Malloy ; Molly ; Singh Harsimran ; Habif Steph ;. (2021). Real-World Patient-Reported Outcomes and Glycemic Results with Initiation of Control-IQ Technology.. *Diabetes technology & therapeutics*, 23(2), pp.120-127. |
| Ravens-Sieberer U and Bullinger M . (1998). Assessing health-related quality of life in chronically ill children with the German KINDL: first psychometric and content analytical results.. *Quality of life research : an international journal of quality of life aspects of treatment and care and rehabilitation*, 7(5), pp.399-407. |
| Rendell Sara, Kosoko-Lasaki Omofosolade and Penny Greg ; Cook Cynthia T; Sharma Apoorva ; Austin William P; Rendell Marc ;. (2013). Improved quality of life in unselected insulin pump-treated children with type 1 diabetes in Eastern Nebraska.. *Journal of diabetes science and technology*, 7(2), pp.579-81. |
| Reviriego J, Millan M D and Millan M ;. (1996). Evaluation of the diabetes quality-of-life questionnaire in a Spanish population. An experience of translation and reliability.. *PharmacoEconomics*, 10(6), pp.614-22. |
| Sikorová Lucie and Valiašková Tatiana . (2020). PSYCHOMETRIC PROPERTIES OF THE CZECH VERSION OF THE TOOL - SCREENING FOR EARLY EATING DISORDER SIGNS (SEEDS-CZ).. *Central European Journal of Nursing & Midwifery*, 11(3), pp.105-112. |
| Siminerio L M, Charron-Prochownik D and Banion C ; Schreiner B ;. (1999). Comparing outpatient and inpatient diabetes education for newly diagnosed pediatric patients.. *The Diabetes educator*, 25(6), pp.895-906. |
| Soni Astha and Ng Sze May. (2014). Intensive diabetes management and goal setting are key aspects of improving metabolic control in children and young people with type 1 diabetes mellitus.. *World journal of diabetes*, 5(6), pp.877-81. |
| Stefaniak Aleksandra A, Zubkiewicz-Kucharska Agnieszka and Matusiak Lukasz ; Noczynska Anna ; Szepietowski Jacek C;. (2020). Itch in Children with Type 1 Diabetes: A Cross-Sectional Study.. *Dermatology and therapy*, 10(4), pp.745-756. |
| Uday S, Campbell F M and Cropper J ; Shepherd M ;. (2014). Monogenic diabetes and type 1 diabetes mellitus: A challenging combination. *Practical Diabetes*, 31(8), pp.327-330. |
| Urqui A C. (2010). Type 1 diabetes and quality of life related with health in children and adolescents. *Diabetes tipo 1 y calidad de vida relacionada con la salud en ninos y adolescentes*, 66(4), pp.244-250. |
| Varni James W and Curtis Bradley H; Abetz Linda N; Lasch Kathryn E; Piault Elisabeth C; Zeytoonjian Andrea A;. (2013). Content validity of the PedsQLTM 3.2 Diabetes Module in newly diagnosed patients with Type 1 diabetes mellitus ages 8-45.. *Quality of life research : an international journal of quality of life aspects of treatment and care and rehabilitation*, 22(8), pp.2169-81. |
| Wagner Julie A, Abbott Gina and Lett Syretta ;. (2004). Age related differences in individual quality of life domains in youth with type 1 diabetes.. *Health and quality of life outcomes*, 2, pp.54. |
| Willems Danielle C M and Joore Manuela A; Nieman Fred H M; Severens Johan L; Wouters Emiel F M; Hendriks Johannes J E;. (2009). Using EQ-5D in children with asthma, rheumatic disorders, diabetes, and speech/language and/or hearing disorders.. *International journal of technology assessment in health care*, 25(3), pp.391-9. |
